# Corruption, accountability, and discretion of procurement officials: An analysis of selection Preferences for Performance-based Evaluation Criteria (PBEC) in PPP procurement

**DOI:** 10.1371/journal.pone.0282542

**Published:** 2023-03-09

**Authors:** Fuguo Cao, Cong Wang

**Affiliations:** 1 School of Law & School of Finance and Taxation, Chinese Academy of Governance for Public Private Partnerships, Central University of Finance and Economics, Beijing, China; 2 School of Finance and Taxation, Chinese Academy of Governance for Public Private Partnerships, Central University of Finance and Economics, Beijing, China; Shenzhen University, CHINA

## Abstract

Performance-based evaluation criteria (PBEC) are vital for selecting high-quality suppliers and achieving a PPP procurement performance. Through theoretical and institutional analysis, we found that the selection of PBEC centered on operations depends on the discretion of the purchaser. However, in an emerging and transforming PPP market, many factors have affected the scientific exercise of the purchaser’s discretion. This means that PPP projects must focus on construction and neglect operation in a certain period. Furthermore, to explore the influencing factors of the definition of PBEC, based on data of 9082 PPP projects between 2009 and 2021 in China, we adopted Ordinary Least Squares to empirically analyze two factors that influence the level of attention that is paid to the operation plan: corruption and accountability. The results indicate that the attention paid to the operation plan significantly increased with the reduction in corruption and the improvement in accountability. Robustness tests demonstrate the robustness of the results. A further heterogeneity analysis shows that the above factors have a more significant impact on non-state demonstration projects and projects with large investments. The contributions of this study are as follows: (1) Theoretically, this paper supplements the relevant research on evaluation criteria and provides new evidence on the impact of corruption and accountability on the defining PBEC. (2) Institutionally, it provides specific paths to limit the discretion of procurement officials when defining evaluation criteria. (3) In practice, it helps procurement officials to scientifically define PBEC and promote the realization of procurement performance.

## 1. Introduction

With the application of PPP in recent years, how to achieve a procurement performance has become an important challenge faced by governments around the world, and has attracted more and more scholars’ attention [1, 2]. As an important way to improve procurement performance [3–6], performance-based procurement has been widely used in jurisdictions such as the United States [7], the United Kingdom [5, 8], the World Bank [9], and the African Development Bank [10]. In performance-based procurement, the contractor is paid only for achieving the agreed results, not for the inputs and activities [11]. Correspondingly, PPP procurement is a typical performance-based procurement. This means that the long-term agreement between the Contracting Authority and the Private Partner for providing a public asset or service, in which the Private Partner bears significant risk and management responsibility, and remuneration is linked to performance [12]. It can be seen that, consistent with performance-oriented procurement, the typical feature of PPP procurement is pay-for-performance, especially emphasizing payment based on operational performance. However, in an immature PPP market in transition, the transition from the traditional infrastructure investment and construction procurement model to the operation-based PPP procurement model faces major challenges. The selection of suppliers based on PBEC is the key to achieving the performance goals and promoting the success of PPP projects.

As an important aspect of performance-based procurement, performance-based evaluation criteria (PBEC) refer to the standards and criteria reflecting the performance goal of the procurement. This is set in procurement documents to evaluate and compare the bids submitted by suppliers, and play a crucial role in achieving procurement performance.

First, PBEC are important to implement output specifications, which refer to the procurement specifications defined based on outputs or outcomes [13]. These are the core of performance-based procurement [14]. This is because the evaluation criteria should be defined based on the procurement specifications according to the requirements of the Guiding Opinions of the Ministry of Finance on Further Strengthening the Management of Government Procurement Demand and Contract Performance Acceptance (Cai Ku [2016] No. 205). Thus, the PBEC is an important measure to achieve procurement performance.

Second, PBEC are an important incentive for potential suppliers to prepare performance-based bid documents. To win the bid, potential suppliers will prepare bids according to the evaluation criteria in the procurement documents; thus, PBEC can guide potential suppliers to prepare bid documents based on performance targets.

Third, PBEC are vital to select suppliers who can achieve a procurement performance [15]. Choosing a quality partner is one of the most important steps in a PPP project [16]. It is not only key to the success of PPP projects [17, 18], but also important to achieving procurement performance [19–22]. In addition, a scientific and reasonable evaluation system is pivotal for choosing partners in government procurement decision-making [18]. Designing scientific evaluation criteria helps select suppliers who can undertake project risks (construction risks, operational risks, etc.), so as to achieve reasonable risk allocation and reduce the risk of renegotiation [23]. The purchaser can select the supplier that best meets the performance objectives and standards following the PBEC, which is announced in advance.

Therefore, in public procurement transformation towards a PPP procurement model, research on PBEC is very important and timely.

Existing research on PPP procurement in China does not pay enough attention to issues in the procurement phase [24]. Among them, Cao & Wang (2022a) [25] and Pu et al. (2020) [26] studied the influencing factors of procurement method selection. Cao & Wang (2022b) [27] studied the determinants of the degree of pay-for-performance in procurement contracts. For evaluation criteria, existing research mainly focuses on how to construct evaluation criteria using the following two aspects: First, regarding the specific content of evaluation criteria, some scholars have constructed an overall evaluation index system. For example, Zhang (2005) [28] identified a set of major evaluation criteria, which includes financial, technical, safety, health, and environmental, and managerial criteria. Furthermore, he identified the relative importance of these criteria when evaluating the private sector in PPP projects. Gan et al. (2018) [29] focused on the importance of five evaluation criteria in the process of supplier selection: construction and operation ability, risk management ability, environmental practices, business value formation, and financial ability. Zhang et al.(2019) [15] focused on the private sectors’ selection criteria for five categories of electric-vehicle charging infrastructure PPP projects: basic ability, management ability, performance of previous performance and credit performance, performance of projects, and sustainable development. However, some scholars have conducted in-depth research on specific selection criteria, such as sustainable supplier selection criteria [30, 31]. Marques and Berg (2011) [23] argue that risk allocation should be given some weight as an award criterion. Second, for research methods, a set of research frameworks was formed with the construction of evaluation indicators and the calculation of indicator weights, such as Gao (2018) [31], Kumaraswamy (2008) [17], etc.

The above research laid a solid foundation for the study of evaluation criteria, although the following void can be noted: the attention to the formation of PBEC and influencing factors used to define PBEC are insufficient. Therefore, this study focuses on addressing the following two questions:

1. How are the PBEC formed in PPP projects?
2. What factors influence the selection of PBEC in PPP projects?

To answer the above two questions, first, this study explores the idea that the generation of evaluation criteria depends on the discretion of procurement officials using a theoretical and institutional analysis. Second, regarding the influencing factors, this study focuses on the impact of corruption and accountability on the selection of PBEC. The reasons for this are as follows: the process used to define the evaluation criteria can be regarded as the process of exercising discretion by the purchaser. As a rational economic person, the purchaser is usually self-interested when making decisions [25]. They evaluate the benefits and costs, and then decide whether or not to engage in rule-breaking behavior [32]. The benefits are usually monetary, and the costs include the likelihood of detection and the severity of sanctions [33]. The former is usually associated with corruption, while the latter is usually associated with accountability. Moreover, the influence of corruption and accountability on the purchaser’s decision-making is also prominent in PPP procurement [25, 34, 35]. Specifically, based on the data of 9082 PPP projects in China from 2009 to 2021, this paper adopts the Ordinary Least Squares to empirically analyze its impact on defining PBEC from the two dimensions of corruption and accountability.

The contributions of this study are as follows: (1) Theoretically, this paper supplements the relevant research on evaluation criteria and provides new evidence on the impact of corruption and accountability on the defining PBEC. (2) Institutionally, it provides specific paths for procurement officials to scientifically exercise discretion when selecting PBEC. (3) In practice, it helps procurement officials to scientifically define PBEC and promote the realization of procurement performance.

This research is arranged as follows: the second section presents the theoretical analysis and research assumptions; the third part is the research design; the fourth part is the empirical results; the fifth part is the discussion; the sixth part is the conclusion.

## 2. Theoretical analysis and research assumptions

### 2.1 Selection of PBEC subject to the discretion of procurement officers

Discussions of discretion in public procurement have long been central to the development of procurement law [36, 37]. Traditional procurement law restricts the discretion, but the fear of discretion has limited the vitality of the procurement rules [38]. Moreover, over-regulation results in purchasing for process rather than value, and this constrains the achievement of the procurement performance. Therefore, the procurement rules now provides more discretion to procurement officials.

For the selection and formulation of evaluation criteria, the procurement law has already established some regulations. However, the extent to which PBEC are defined and how much weight PBEC occupy in the entire evaluation criteria system still depends on the discretion of the purchaser. For example, in the government procurement rules, the ***UNCITRAL Model Law on Public Procurement*** and ***Government Procurement Law*** in China all require that the selection of evaluation criteria relates to the subject matter of the procurement and is announced in advance. The unannounced indicators cannot be used as evaluation criteria. In the PPP procurement rules, the ***UNCITRAL Model Legislative Provisions on Public-Private Partnerships*** stipulates the importance of operations in the evaluation criteria. PPP procurement rules in China put forward relatively specific requirements regarding the definition of evaluation criteria and required the comprehensively evaluation of various factors to select the best supplier. The State Council, the Ministry of Finance (MoF), the National Development and Reform Commission and other departments have issued documents requiring PPP projects to comprehensively consider the following criteria: technical capabilities, management experience, financial strength, business performance, professional qualifications, credit status, investment and financing capabilities, contract performance capabilities, quotations and other factors. However, the downside is that different documents issued by different departments place different levels of emphasis on the evaluation criteria. Even different documents issued by the same department have different regulations regarding evaluation criteria. When facing a complex environment with vague or conflicting rules, administrators inevitably show varying degrees of discretion [39, 40]. Therefore, procurement officials show greater discretion when defining the evaluation criteria. A small number of these rules are given de facto priority, and which rules are followed is subject to the officials’ discretion [41].

In PPP procurement, defining PBEC centered on operations is the key to selecting high-quality suppliers and achieving procurement performance. The success of PPP projects depends on the design of operational performance and the selection of suppliers based on operational performance criteria. The emphasis on operational performance is an essential feature of PPP procurement, which is enough to provide it with a value-for-money advantage over traditional procurement models. Specifically, PPP is an innovative model where the public sector and the private sector provide public goods and services through cooperation under the principles of risk-sharing and benefit-sharing. Its core meaning is the high-quality supply of public goods and services [42]. Additionally, the government and private sector establish output targets in the contract, and the scope of project cooperation should extend from the construction of traditional infrastructure to the supply of core services [42]. In addition, the private sector participates in the entire process of investing, constructing, managing and operating in PPP projects. From the perspective of the long life-cycle of a PPP project, compared with the fixed assets formed during the construction period, the operation effect can better represent the outputs and results of the project. Chen and Hu (2020) [43] also pointed out the operation capability should be the core of the value-for-money evaluation of PPP projects. Besides, the PPP-related policies issued by various departments in China also emphasize the importance of operation.

However, in an emerging and transforming PPP market, many factors affect the scientific exercising of the purchaser’s discretion. This means that PPP projects often focus on construction and neglect operation in a certain period [43]. The separation of construction and operation means that cooperation between the public and private sectors only occurs at the construction stage for infrastructure. This inhibits the quality improvement and synergy effect of the PPP model [42], and restricts the realization of procurement performance. The stronger emphasis on the construction criteria may be because the public sector is more focused on the economic aspect (cheapest solution output) in the value-for-money assessment rather than efficiency (highest quality) and effectiveness (outcome achievement), which are more related to long-term benefits. The emphasis on construction criteria may also result from improper risk allocation where substantial (operational) risk may not be properly transferred to the private partner, an assertion in line with Marques and Berg(2011) [23] who argue that risk allocation should be given some weight as an award criterion. Therefore, to optimize the exercise of discretion and promote the realization of PPP procurement performance, it is important to explore the factors that could influence the purchaser’s exercising of discretion when selecting evaluation criteria.

### 2. 2 Theoretical analysis of factors influencing the selection of PBEC

#### 2. 2. 1 Corruption

Corruption is one of the main obstacles to sustainable socio-economic and political development in developed, developing and emerging economies. Not only does it increase inequality, it also reduces efficiency [44].

In public procurement, corruption can affect the discretion of procurement officials. For example, procurement officials have some discretion in the selection of suppliers. Potential suppliers may collude with procurement officials in order to win bids, thereby causing procurement officials to offer more opportunities to certain suppliers [45]. Especially in the context of the tenure system for officials in China, tenure shows uncertainty in time and space. At this time, local officials are only temporarily in charge of local affairs, and opportunistic behavior tends to be serious. When interest incentives appear, local officials are often eager to grab short-term benefits [46]. Decision-makers tend to give more weight to the performance closer to the present time at the expense of long-term performance. In addition, the procurement sector has long been considered the most vulnerable sector to corrupt activities [35, 47]. Therefore, in public procurement, the decisions of procurement officials are more susceptible to corruption.

In addition, in PPP procurement, corruption is an important factor affecting the success of the project and acts on the entire life-cycle of the project [48]. The magnitude of many PPP projects, their typically long duration, and the need for constant interaction between government officials, agents of the contracting authority and employees or agents of the private partner may encourage and create innumerable opportunities for bribery, extortion or other corrupt practices [49]. In PPP projects, purchasers with a corrupt mentality often hope to choose a private sector that can meet their corrupt needs under the discretionary space allowed by law, and then favor specific suppliers when designing evaluation criteria.

In PPP procurement, corruption causes purchasers to pay more attention to construction than operation when selecting evaluation criteria. The reasons are as follows: (1) In PPP projects, for suppliers, compared with the operation period, the project construction period has greater profit space, stronger stability, and shorter time to obtain benefits. Therefore, construction-focused suppliers are more motivated than operation-focused suppliers to bribe procurement officials to win bids. This has a higher probability of success in areas with higher levels of corruption. For purchasers with a corrupt mentality, facing the temptation of corrupt interests, it is easier to attach importance to construction and despise operation when formulating evaluation standards. (2) In areas with a higher degree of corruption, purchasers often pay less attention to the realization of long-term performance goals in PPP procurement due to corrupt interests. As more emphasis is placed on operational effectiveness, there is a greater possibility of corruption being discovered. Therefore, the higher the degree of corruption, the more emphasis the purchaser places on construction, while less emphasis is placed on operation.

**Hypothesis 1: In areas with higher levels of corruption, less emphasis is placed on operating plans in the evaluation criteria for PPP projects**.

#### 2. 2. 2 Accountability

The institutional environment is an important factor affecting the performance of PPP procurement [50]. In general, the continued growth and mobilization of private investment in infrastructure through PPPs is largely dependent on key enabling institutional factors and regulatory conditions within a country [1]. Opara et al. (2017) [51] also show that a favorable policy environment is an important prerequisite for the successful implementation of PPPs.

When defining evaluation criteria, the purchaser inevitably has a risk-averse preference while exercising their discretion. When facing performance-based normative requirements, the purchaser’s decision-making is more inclined to follow the rules and define PBEC. The definition of evaluation criteria is the result of the purchaser’s preferred decision. As a rational economic person, the purchaser usually has a self-interested mentality when making decisions [25]. As they are motivated by their own safety and self-preservation, they are constantly driven to seek a "haven" of immunity in the bureaucratic logic of doing things by the rules [52]. When the likelihood of rule violations being detected or the severity of sanctions increases, purchasers tend to act more in line with the rules [33].

Since the second half of 2017, the CPC Central Committee and the State Council have given the prevention and resolution of major risks more prominence. The National Financial Work Conference, the Central Economic Work Conference and the State Council executive meeting have all made clear arrangements for the strictly control of local government debt and require the rectification of irregularities in the PPP market [53]. PPP has entered a period of strict regulation. Furthermore, the MoF and other departments intensively issued a series of documents to promote the normative development of PPP procurement, focusing on solving the widespread problem of emphasizing construction and ignoring operation in practice [43]. Furthermore, these rules closely link payment with construction performance and operational performance. The central policy also stipulates the means of illegal sanctions, including reviewing and rectifying projects in the project management database, standardizing the standards used to enter the database, and public condemnation. The above measures have affected the purchaser’s discretion when selecting evaluation criteria. To avoid accountability and minimize their losses, buyers are more inclined to regulate their behavior under the constraints of these rules [25]. Therefore, the more emphasis is placed on quality and effectiveness in PPP procurement rules, the more likely procurement officials are to conduct result-based procurement. They also find it easier to restrain their behavior and develop the project in a more standardized and high-quality direction.

**Hypothesis 2: The stronger the accountability, the more emphasis will be placed on the operation plan in the evaluation criteria for PPP projects**.

## 3. Research design

### 3. 1 Sample data and sources

From January 1, 2009, to March 21, 2021, there were a total of 9082 projects in the procurement and execution stages in the Chinese PPP project management database. After we manually read the evaluation criteria in the procurement documents, the number of valid projects with construction or operation plans was 5653.

### 3. 2 Model

#### 3. 2. 1 Dependent variable and proxy variable

The dependent variable is the importance attached by the purchaser to the operation plan in the PPP project evaluation criteria. This study used the proportion of the score of the operation plan to the sum of the scores of the construction plan and the operation plan to measure the explained variables. The higher the proportion of the score of the operation plan, the more attention the purchaser attaches to the operation plan and the less attention they attach to the construction plan. This better allows PPP project to achieve the necessary operation effect and procurement performance.

The above data derived from the evaluation criteria in the PPP project procurement documents in the project management database of the PPP Center of MoF.

#### 3. 2. 2 Independent variables and proxy variables

The independent variables fall into two categories: corruption and accountability. The specific indicators and data sources are as follows:

For corruption, we chose the number of crimes regarding corruption and malfeasance as the measure. The reasons for this are as follows: Raymond & Roberta (2002) [54] used the number of civil servants convicted of abuse of power as a measure of the level of corruption in US states and obtained satisfactory results. They pointed out that the crime rate of civil servants’ abuse of power is a reasonable indicator of the true level of corruption. Zhou & Tao (2009) [55] used the total number of corruption cases in the region as an indicator of the degree of corruption. Our paper drew on the above research and used the number of indictments filed by the procuratorate for crimes of corruption and malfeasance as an indicator to measure the degree of corruption. The data derived from the PKULAW database.

For accountability, we formed a distinction based on the release of the Notice on Regulating the Management of the Project Database of the PPP Comprehensive Information Platform (Caibanjin [2017] No. 92). On November 10, 2017, the MoF issued the Caibanjin [2017] (No. 92), which requires each provincial finance department to conduct a centralized review of their project management database. The document requires that projects that only involve engineering construction but have no operational content not be put into storage, and these projects shall not be responsible for expenditure through financial budgets according to the Notice of the Ministry of Finance on Regulating the Operation of the Public–Private Partnership (PPP) Comprehensive Information Platform (Caijin [2015] No. 166). The release of this document released a signal to regulate the development of PPP and triggered the most severe "normative effect" in the history of PPP procurement [53]. Accountability also increased as a result. Therefore, this study took November 10, 2017 as the boundary. The accountability of PPP projects initiated before this date was weak, and recorded as 0. PPP projects initiated after this date have stronger accountability and were recorded as 1. The project initiation time data derived from the Wind database.

#### 3. 2. 3 Control variables

For control variables, to control the impact of other project characteristics on the evaluation criteria, this study controlled the duration, industry type, demonstration type, investment, return model, region, operation model and procurement method. The model considers most of the characteristics of PPP projects. There may be other factors relevant to the definition of evaluation criteria but, due to data limitations, we could not include all possible factors. The above data derived from the Wind database.

The regression model is as follows:

$$Op_{m}=\alpha_{m}+\beta_{m1}X_{i1}+\cdots +\beta_{mk}X_{ik}+\varepsilon_{m}$$ (1)

The independent variables were X_i1_⋯X_ik_ respectively. The coefficients were as follows: α_m_ is the intercept term, ε_m_ is the residual. To determine multicollinearity, a Variance Inflation Factor (VIF) test was performed. We found that the VIF values of all variables were less than 2, which indicates that the model does not have serious multicollinearity problems. The variable description is shown in Table 1.

**Table 1:** Description of variables.

Type	Variable	Description	Source
Dependent variable	The degree of emphasis on the operating plan	Continuous variable, the degree of emphasis on the operation plan = the score of the operation plan in the contract award standard / (the score of the construction plan + the score of the operation plan)	PPP database of PPP Center
Independent variables	Corruption	Continuous variable, number of indictments for corruption and malpractice offences, logarithm	PKULAW database
	Accountability	Dummy variable, PPP projects initiated before November 10, 2017 = 0, PPP projects initiated after November 10, 2017 = 1	Wind database
Control variables	Duration	Continuous variable, cooperation period of PPP	Wind database
	Industry type	Dummy variables, traditional infrastructure projects = 0, non-traditional infrastructure projects = 1	
	Demonstration type	Dummy variable, national demonstration project = 1, others = 0	
	Investment	Continuous variable, PPP project investment amount, logarithm	
	Return model	Multi-valued variable, government payment = 1, Feasibility gap subsidy = 2, user payment = 3	
	Region	Multi-valued variable, eastern region = 1, central region = 2, western region = 3	
	Operation model	Multi-valued variable, BOT = 1, BOO = 2, TOT = 3, ROT = 4, MC = 5, O&M = 6, others = 7, BOT+TOT = 8, BOO+TOT = 9	
	Procurement method	Dummy variable, tendering method = 0, negotiation method = 1	PPP database of PPP Center

## 4. Empirical results

### 4. 1 Descriptive statistics

**Fig 1:**
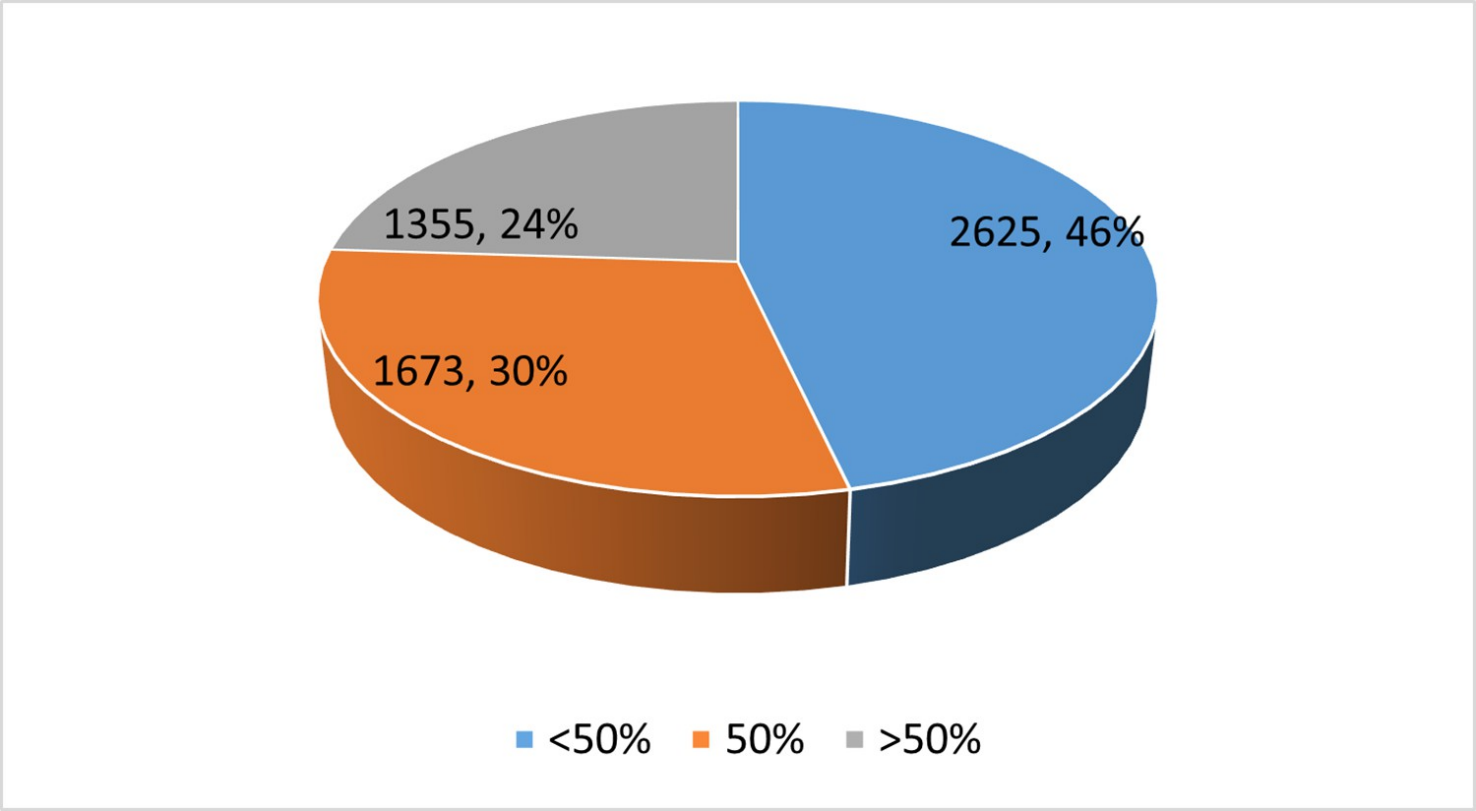
The number and proportion of PPP projects corresponding to the proportion of different operation plans.

Fig 1 shows the number and proportion of PPP projects corresponding to the proportion of the different levels of attention to the operation plan. We can see that, in the evaluation criteria of PPP projects, 46% of the projects have a lower score for the operation plan than the construction plan, 30% of the projects have the same score for the operation plan and the construction plan, and only 24% of projects scored higher for the operation plan than the construction plan. This indicates that the emphasis on the operation plan is still insufficient in the practice of PPP procurement. Table 2 presents descriptive statistics of corruption. Table 3 shows the descriptive statistics of the importance attached to the operation plan in the PPP project evaluation criteria under different accountability levels. This reveals that, with the increase in accountability, the buyer’s average emphasis on the operation plan gradually increases. However, descriptive statistics are only a preliminary description of variables. To obtain an accurate relationship between independent variables and a dependent variable, it is necessary to carry out standardized regression.

**Table 2:** Descriptive statistics of corruption.

Variable	Obs	Mean	Std. Dev.	Min	Max
Corruption	5, 653	4003. 348	1504. 569	285	6753

**Table 3:** Descriptive statistics of the degree of emphasis on the operation plan in the evaluation criteria for PPP projects under different accountability levels.

Variable	Obs	Mean	Std. Dev.	Min	Max
Week accountability	4, 350	0. 4579144	0. 2149801	0	1
Strong accountability	1, 303	0. 5043753	0. 198357	0	1

### 4. 2 Regression results

Table 4 was obtained by performing ordinary least squares regression on Eq (1). The following results were found:

First, for corruption, when controlling for other variables in areas with higher levels of corruption, PPP project purchasers place less emphasis on operating plans in the evaluation criteria. Hypothesis 1 is verified.

Second, in the dimension of accountability, while controlling for other variables, the stronger the accountability, the more emphasis the purchaser attaches to the operation plan in the evaluation criteria. Hypothesis 2 is verified.

**Table 4:** Baseline regression results.

Variables	Coef.	Std. Err.
Corruption (log)	-0. 0185***	(0. 00600)
Accountability	0. 0465***	(0. 00651)
Duration	0. 00440***	(0. 000427)
Industry type	0. 0194***	(0. 00674)
Investment (log)	-0. 0303***	(0. 00217)
Return model	0. 00958*	(0. 00547)
Demonstration type	0. 0161*	(0. 00940)
Regions	0. 00107	(0. 00343)
Operation model	0. 0105***	(0. 00125)
Procurement method	0. 0586***	(0. 00810)
Constant	0. 784***	(0. 0570)
Observations	5, 645	
R-squared	0. 093	

Robust standard errors in parentheses

*** p<0. 01

** p<0. 05

* p<0. 1

### 4. 3 Robustness test

#### 4. 3. 1 Reduce the sample size

To test the robustness of the benchmark regression results, we performed regression after adjusting the sample size. After removing the agriculture [38], pension [49], sports [46], and social security [14] projects, Table 5 was obtained after regression. This shows that the regression results are consistent with the benchmark regression results. This demonstrates the robustness of the benchmark regression results.

**Table 5:** Regression results with reduced sample size.

Variables	Coef.	Std. Err.
Corruption (log)	-0. 0196***	(0. 00611)
Accountability	0. 0467***	(0. 00658)
Duration	0. 00446***	(0. 000432)
Industry type	0. 0173**	(0. 00693)
Investment (log)	-0. 0304***	(0. 00220)
Return model	0. 00785	(0. 00553)
Demonstration type	0. 00965	(0. 00949)
Regions	0. 00112	(0. 00346)
Operation model	0. 0107***	(0. 00126)
Procurement method	0. 0584***	(0. 00823)
Constant	0. 798***	(0. 0581)
Observations	5, 498	
R-squared	0. 093	

Robust standard errors in parentheses

*** p<0. 01

** p<0. 05

* p<0. 1

#### 4. 3. 2 Replacing proxy variables

Further, we used the method of replacing proxy variables for robustness testing. Fiscal transparency in a region is associated with levels of corruption. Scholars such as Li (2016) [56], Haque et al. (2019) [57], and Du (2020) [58] have proved, through empirical research, that the higher the fiscal transparency, the lower the level of corruption, and the International Monetary Fund believes that fiscal transparency is an important prerequisite for ensuring integrity. Therefore, we replaced the corruption with fiscal transparency, and Table 6 was obtained after regression. The results demonstrate that the higher the financial transparency, the higher the importance that the purchaser places on the operation plan in the evaluation criteria. This finding further validates the robustness of the benchmark regression results.

**Table 6:** Regression results of substitution proxy variables.

Variables	Coef.	Std. Err.
Fiscal transparency	0. 000671***	(0. 000205)
Accountability	0. 0262***	(0. 00767)
Duration	0. 00439***	(0. 000446)
Industry type	0. 0172**	(0. 00700)
Investment (log)	-0. 0315***	(0. 00224)
Return model	0. 0103*	(0. 00567)
Demonstration type	0. 0202**	(0. 00944)
Regions	0. 00675*	(0. 00365)
Operation model	0. 0103***	(0. 00128)
Procurement method	0. 0590***	(0. 00816)
Constant	0. 606***	(0. 0286)
Observations	5, 150	
R-squared	0. 094	

Robust standard errors in parentheses

*** p<0. 01

** p<0. 05

* p<0. 1

## 5. Heterogeneity analysis

Through the above analysis, we found that both corruption and accountability significantly affect the purchaser’s attention to the operating plan in the evaluation criteria. To further observe the differences between the above impacts in different types of projects, we conducted a heterogeneity analysis focusing on the two aspects of demonstration type and investment. The regression results are shown in Table 7.

**Table 7:** Heterogeneity analysis regression results.

	Demonstration type		Investment	
	(1)	(2)	(3)	(4)
VARIABLES	National demonstration projects	Non-national demonstration projects	804. 09–61000 million yuan	61013–8980000 million yuan
Corruption (log)	0. 0134	-0. 0237***	-0. 00979	-0. 0263***
	(0. 0156)	(0. 00653)	(0. 00814)	(0. 00868)
Accountability	-0. 0111	0. 0471***	0. 0187**	0. 0747***
	(0. 0231)	(0. 00655)	(0. 00924)	(0. 00900)
Duration	0. 00565***	0. 00422***	0. 00555***	0. 00324***
	(0. 00125)	(0. 000454)	(0. 000596)	(0. 000627)
Industry type	0. 0390*	0. 0172**	0. 0108	0. 0269***
	(0. 0235)	(0. 00704)	(0. 0100)	(0. 00914)
Investment (log)	-0. 0262***	-0. 0307***	-0. 0330***	-0. 0328***
	(0. 00683)	(0. 00230)	(0. 00554)	(0. 00452)
Operation model	0. 0170	0. 00834	0. 00776	0. 0118
	(0. 0160)	(0. 00584)	(0. 00758)	(0. 00789)
Demonstration type	-	-	-0. 00435	0. 0349***
			(0. 0146)	(0. 0121)
Regions	-0. 0176	0. 00321	0. 0138***	-0. 00976**
	(0. 0112)	(0. 00361)	(0. 00494)	(0. 00480)
Operation model	0. 0110***	0. 0105***	0. 0144***	0. 00625***
	(0. 00351)	(0. 00133)	(0. 00196)	(0. 00158)
Procurement method	0. 0504**	0. 0584***	0. 0531***	0. 0643***
	(0. 0198)	(0. 00889)	(0. 0109)	(0. 0120)
Constant	0. 465***	0. 836***	0. 705***	0. 913***
	(0. 153)	(0. 0618)	(0. 0914)	(0. 0957)
Observations	598	5, 047	2, 823	2, 822
R-squared	0. 107	0. 093	0. 098	0. 080

Robust standard errors in parentheses

*** p<0. 01

** p<0. 05

* p<0. 1

First, in terms of demonstration types, compared with national demonstration projects, the discretion of purchasers in non-state demonstration projects is more affected by corruption and accountability. That is to say, the increase in corruption makes the purchaser in the non-state demonstration project pay less attention to the operation plan in the evaluation criteria but has no significant impact on the purchaser’s discretion in the state demonstration project. In addition, the increased accountability leads the purchaser in the non-state demonstration project to pay more attention to the operation plan in the evaluation criteria, but has no significant impact on the purchaser in the national demonstration project.

Second, regarding investment, the purchaser’s discretion is more significantly affected by corruption and accountability in projects with large amounts of investment. Specifically, the increase in corruption causes the purchaser to pay less attention to the operation plan in the evaluation criteria of PPP projects with large levels of investment, but has no significant impact on the purchaser in PPP projects with low levels of investment. For accountability, regardless of the amount of investment, with the increase in accountability, the purchaser’s preference for the operation plan in evaluation criteria significantly increases. This is consistent with the benchmark regression results. Additionally, the discretion of the purchaser with a larger project investment is higher, and is more significantly affected by the accountability.

## 6. Discussion and Implication

### 6. 1 Discussion

The evaluation criteria are the basic basis for determining the winning supplier, and their emphasis on the operation plan directly determines the quality and efficiency of the project operation. This is an effective way to solve the problem in which construction is emphasized and operation is ignored, thereby promoting the realization of PPP performance. However, according to statistics, in China’s PPP procurement practice, only 24% of projects place more higher weight on the operation plan in the evaluation criteria than the construction plan. This shows that the emphasis on operation in PPP projects evaluation criteria is still insufficient. Therefore, finding the influencing factors that affect the exercise of discretion by the purchaser when selecting evaluation criteria is crucial to optimize the exercise of discretion and achieve PPP procurement performance.

This study empirically analyzes the factors that affect officials’ preferred evaluation criteria from the two dimensions of corruption and accountability. The research shows that the purchaser’s attention on the operation plan is affected by corruption and accountability.

First, the degree of corruption affects the attention that the purchaser pays to the operation plan in the evaluation criteria. Procurers in regions with higher levels of corruption place less emphasis on the operation plan in the evaluation criteria. This confirms the view of Chan et al. (2011) [59] that corruption affects the success of PPP projects from the perspective of the definition of evaluation criteria. In addition, the results of the heterogeneity analysis indicate that, compared with national demonstration projects, the discretion of purchasers in non-state demonstration projects is more significantly affected by corruption. A possible reason for this is that the national demonstration projects undergo a higher-standard recommendation and selection process. Their purpose is to guide the normal operation of PPP procurement and to promote empirical models. To this end, the MoF issued some targeted documents specifically for the standardized operation of demonstration projects and regulated demonstration projects through measures such as strengthening tracking management, strengthening information disclosure, and improving long-term management mechanisms. This makes demonstration projects more regulated and leaves less room for corruption than non-demonstration projects. As a result, demonstration projects are less susceptible to corruption. Additionally, PPP projects with higher investment are more significantly affected by corruption than PPP projects with lower investment. The possible reason for this is that, for procurement officials, higher-investment projects have more room for corrupt gains. Therefore, they are also more susceptible to the influence of corrupt psychology.

Second, accountability affects the attention that buyers place on the operation plan in the evaluation criteria. The stronger the accountability, the more attention the purchaser attaches to the operation plan in PPP procurement. This result confirms the view of Zhang et al.(2015) [50], Casady(2020) [1] and others regarding defining the evaluation criteria of PPP projects: the institutional environment is critical to PPP procurement performance. This also validates the findings of Cao & Wang (2022a) [25], who stated that risk aversion affects the discretion of purchasers in PPP procurement. Further, the results of the heterogeneity analysis evidence that non-state demonstration projects are more significantly affected by accountability than state demonstration projects. The possible reasons for this are that the national demonstration projects are subject to a higher degree of supervision and stricter requirements, and the procurement behavior of the purchasers is usually more standardized. However, the quality of non-national demonstration projects is uneven, the degree of supervision is relatively weak, and buyers often have more irregular procurement behaviors. Therefore, when accountability suddenly increases, the influence of policy on the behavior of purchasers in non-state demonstration projects is more pronounced.

### 6. 2 Implication

To optimize the purchaser’s discretion and achieve PPP procurement performance, this study provides us with the following suggestions.

First, at the institutional level, the rules and procedures for contract awarding should be optimized to to avoid abuses of discretion by procurement officials. The ambiguous and contradictory rules on evaluation criteria in the current government procurement system and PPP procurement system give purchasers some discretion, and the PBEC depend on the purchaser’s decision. In contrast to PPP legislation at the international level, the ***UNCITRAL Model Legislative Provisions on Public-Private Partnerships*** stipulates the importance of operations in the evaluation criteria. This requires that the criteria for reviewing and comparing the technical elements of proposals should include operational feasibility, service quality and measures to ensure service continuity. The above rules limit the discretion of the purchaser, and help the purchaser to fully consider the operation and service continuity when defining evaluation criteria. In addition, the ***UNCITRAL Legislative Guide on Public-Private Partnerships (2019)*** states that transparent rules and procedures limit the exercise of discretion. Discretion should be monitored and challenged where necessary. The above rules provide a reference for further improvements in China’s contract award rules. Given the above, to avoid abuses of discretion by procurement officials, the following measures can be taken: (1) Formulating PBEC in the ***Government Procurement Law*** to provide a higher-level legal basis for defining PBEC. (2) Clarifying the importance of operation in the evaluation criteria in PPP procurement rules, then providing a specific basis for the definition of PBEC. (3) Optimizing the rules of contract award procedures to make the contract award procedures more transparent.

Second, we should prevent corruption and reduce the influence of corruption on the purchaser’s discretion, and increase the purchaser’s attention to the operation plan in the evaluation criteria. In the construction industry, the impact of corruption on industry and the public is very easy to detect. This includes the shortening of the life of buildings, the collapse of buildings, taking of human lives, etc. [60–62]. It is necessary to ensure that officials of the contracting authority do not directly or indirectly benefit from the project or through dealings with private sectors, and that private sectors should not exercise undue influence over any officials involved in the design, selection, implementation or management of the project. To reduce the level of corruption, the following measures can be taken: (1) Establishing an effective punishment system while avoiding conflicts of interest. The ***UNCITRAL Legislative Guide on Public-Private Partnerships (2019)*** states that, to guard against corruption by government officials, including employees of the contracting authorities, the host country should have an effective system of sanctions in place, and conflicts of interest should also be avoided. (2) Strengthening public participation in the procurement process. The active participation of the public helps to curb corruption to the greatest possible extent [63]. (3) Strengthening the supervision of projects with high investment and non-state demonstration projects.

Third, we should strengthen accountability, thereby increasing the purchasers’ emphasis on the operation plan in the evaluation criteria. For example, we could rectify the PPP projects that ignore the operation plan in the evaluation criteria, hold the relevant procurement officials responsible for setting the evaluation criteria accountable, and then motivate the purchaser to define the PBEC.

Finally, we should further strengthen the demonstration effect of national demonstration projects, give full play to the normative and leading role of demonstration projects, promote the application of an excellent management experience of demonstration projects, and then promote the higher-quality development of projects in different demonstration categories.

## 7. Conclusion

The performance-based evaluation criteria (PBEC) are vital for selecting high-quality suppliers and achieving PPP procurement performance. Through theoretical and institutional analysis, we found that the selection of PBEC centered on operations depends on the discretion of the purchaser. However, in an emerging and transforming PPP market, many factors affect the purchaser’s discretion. This means that focusing on construction and neglecting operation in a certain period is a common proble in PPP projects. Moreover, purchasers still do not pay enough attention to the operation plan in the evaluation criteria during PPP procurement in China. Therefore, this study first analyzed the selection of evaluation criteria, a process in which the purchaser exercises their discretion, which is affected by many factors. Second, using 9082 PPP projects in China, we empirically analyzed the effect of corruption and accountability on the procurement officials’ definition of PBEC. The study found that procurers in regions with less corruption place more emphasis on operational plans in the evaluation criteria. In addition, the stronger the accountability, the more emphasis the purchasers place on operational plans in evaluation criteria.

The contributions of this study are mainly in three aspects:

The above findings have important policy implications: (1) the rules and procedures for selecting suppliers should be optimized to prevent procurement officials from abusing their discretion. (2) Corruption should be controlled to reduce the impact of corruption on purchasers’ discretion. (3) Accountability should be strengthened to encourage purchasers to pay attention to the operation plan. (4) The demonstration effect of national demonstration projects should be strengthened.

These conclusions also have important academic value. This study focuses on the important position of the operation plan in defining the PBEC, and analyzes the impact of corruption and accountability on the purchaser’s PBEC preferences through empirical research. This complements the relevant research on evaluation criteria in PPP procurement and explores the factors influencing the definition of evaluation criteria.

Besides, this research can fundamentally solve the problem of "focusing on construction and neglecting operation" in PPP practice, and promote the realization of PPP procurement performance.

The insufficiencies of this study are as follows: (1) It focuses on the influencing factors of the design of evaluation criteria in the procurement stage, while ignoring the issues in the contract performance stage, such as renegotiation. (2) It only focuses on the importance of the operating plan in the evaluation criteria, and does not pay attention to other evaluation criteria. For example, in PPP procurement, other relevant factors (e.g. technical capabilities, management experience, financial strength, business performance, professional qualifications, credit status, investment and financing capabilities, contract performance capabilities, quotations, and other factors) need to be reviewed, other than the operating plan. However, operating scenarios are an even more important aspect of PBEC. (3) It does not focus on other influencing factors besides accountability and corruption. In the future, we can explore PBEC and its influencing factors from other perspectives.

## Supporting information

S1 Dataset (XLSX)
